# Force coordination distinguishes epithelial and mesenchymal modes of collective chemotaxis

**DOI:** 10.1083/jcb.202507211

**Published:** 2026-01-21

**Authors:** Jorge Diaz, Roberto Mayor

**Affiliations:** 1 Department of Cell and Developmental Biology, University College London, London, UK; 2 Center for Integrative Biology, Faculty of Sciences, Universidad Mayor, Santiago, Chile

## Abstract

Collective cell migration is essential for development and tissue homeostasis and plays a central role in pathological processes such as tumor metastasis. While extensively studied in epithelial cells, collective migration is also observed in mesenchymal cells, though the mechanistic similarities and differences between these modes remain unclear. Here, we use neural crest (NC) cells to investigate collective chemotaxis in epithelial and mesenchymal states within the same lineage. Mesenchymal NC clusters migrate collectively toward the chemoattractant SDF-1 through rear-directed contractility of supracellular actomyosin cables and polarized front-edge protrusions. In contrast, epithelial NC cells exhibit polarized cryptic protrusions and increased active Rac1 localization at E-cadherin–mediated junctions. During epithelial chemotaxis, traction forces originate from internal cell–cell junctions, whereas in mesenchymal clusters, they remain peripheral. Our findings reveal that mesenchymal collective chemotaxis relies on supracellular force coordination, while epithelial chemotaxis depends on force generation by individual cells within the collective.

## Teaser

The authors show that epithelial and mesenchymal cells navigate chemical cues via fundamentally different force-generating mechanisms. Whereas epithelial groups rely on forces produced at internal junctions, mesenchymal clusters depend on coordinated supracellular contractions, offering new insight into how diverse cell types achieve collective movement during development and disease.

## Introduction

During embryogenesis, wound repair, and cancer invasion, cells often migrate together while maintaining physical contact and coordinating their behavior. This process, known as collective migration, can occur in different structural contexts, from cohesive epithelial sheets to more flexible clusters with mesenchymal characteristics (Friedl and Gilmour, 2009; Rørth, 2009; Haeger et al., 2015). While some groups use leader cells and features associated with epithelial organization, others move collectively without clear front cells, relying instead on local interactions and coordinated protrusions (Mayor and Etienne-Manneville, 2016; Rørth, 2009). These behaviors show that a single architecture does not define collective migration, but rather the ability of the group to move more efficiently than individual cells (Haeger et al., 2015; Trepat and Sahai, 2018; Ladoux and Mège, 2017).

The cephalic neural crest (NC) is a well-established model of mesenchymal collective migration. NC cells migrate as clusters guided by chemotactic signals such as the stromal cell–derived factor 1 (SDF1), using mechanisms like contact inhibition of locomotion, co-attraction, and planar cell polarity (Theveneau et al., 2010; Carmona-Fontaine et al., 2008). A key feature of these clusters is a contractile supracellular actomyosin cable at the rear of the cluster, which generates directional movement through pulses of contraction, a mechanism described as “rear-wheel drive” chemotaxis (Shellard et al., 2018; Shellard and Mayor, 2019). This structure has been shown to contribute to directional chemotaxis in mesenchymal NC clusters. However, it is still unknown whether such supracellular organization is required in all contexts or whether alternative mechanical strategies can support guided migration when cell–cell adhesion is altered. Cell migration is often linked to a decrease in epithelial adhesion molecules and the acquisition of mesenchymal traits, consistent with total or partial epithelial-to-mesenchymal transition (EMT) (Lamouille et al., 2014; Nieto, 2013; Scarpa et al., 2015). However, several studies have shown that E-cadherin junctions can persist in migrating cells, including during epithelial repair, branching morphogenesis, and carcinoma invasion (Revenu and Gilmour, 2009; Shamir and Ewald, 2015; Campbell and Casanova, 2016; Cheung et al., 2013; Balasubramaniam et al., 2021; Campàs et al., 2024; Gupta and Yap, 2021; Labernadie et al., 2017; Padmanaban et al., 2019; Sonam et al., 2023). E-cadherin is expressed in *Xenopus* cephalic NC cells at early stages, and its sustained expression has been shown to promote epithelial-like properties and limit cell dispersion under baseline conditions in the absence of directional cues (Scarpa et al., 2015). Consistent with this, its function is required for proper NC migration (Huang et al., 2016), reinforcing its early role in collective movement. In line with this, studies in chick embryos have shown that E-cadherin is robustly maintained during the early phases of cranial NC migration, highlighting its role in supporting cohesive collective movement (Dady et al., 2012; Rogers et al., 2013; Rogers et al., 2018). These findings raise the possibility that, in specific contexts, NC cells might migrate collectively within embryonic streams while retaining epithelial features. Whether this organization can support chemotactic responses and enable directional guidance remains an open and relevant question. To address this, we investigated how epithelial organization, induced by sustained E-cadherin expression, affects the chemotactic migration of cephalic NC cells in *Xenopus laevis*. By combining live imaging, quantitative analysis of protrusions, and traction force microscopy (TFM), we examined how changes in cell–cell adhesion alter polarity, coordination, and force transmission during collective migration. In addition, we compared this epithelial collective chemotaxis with the collective chemotaxis of mesenchymal cells. This approach reveals an alternative mode of chemotaxis that challenges the classical mesenchymal paradigm and highlights the plasticity of collective migration strategies in embryonic cells.

## Results

### E-cadherin–positive NC cells initiate migration while retaining epithelial features

**Figure 1.**
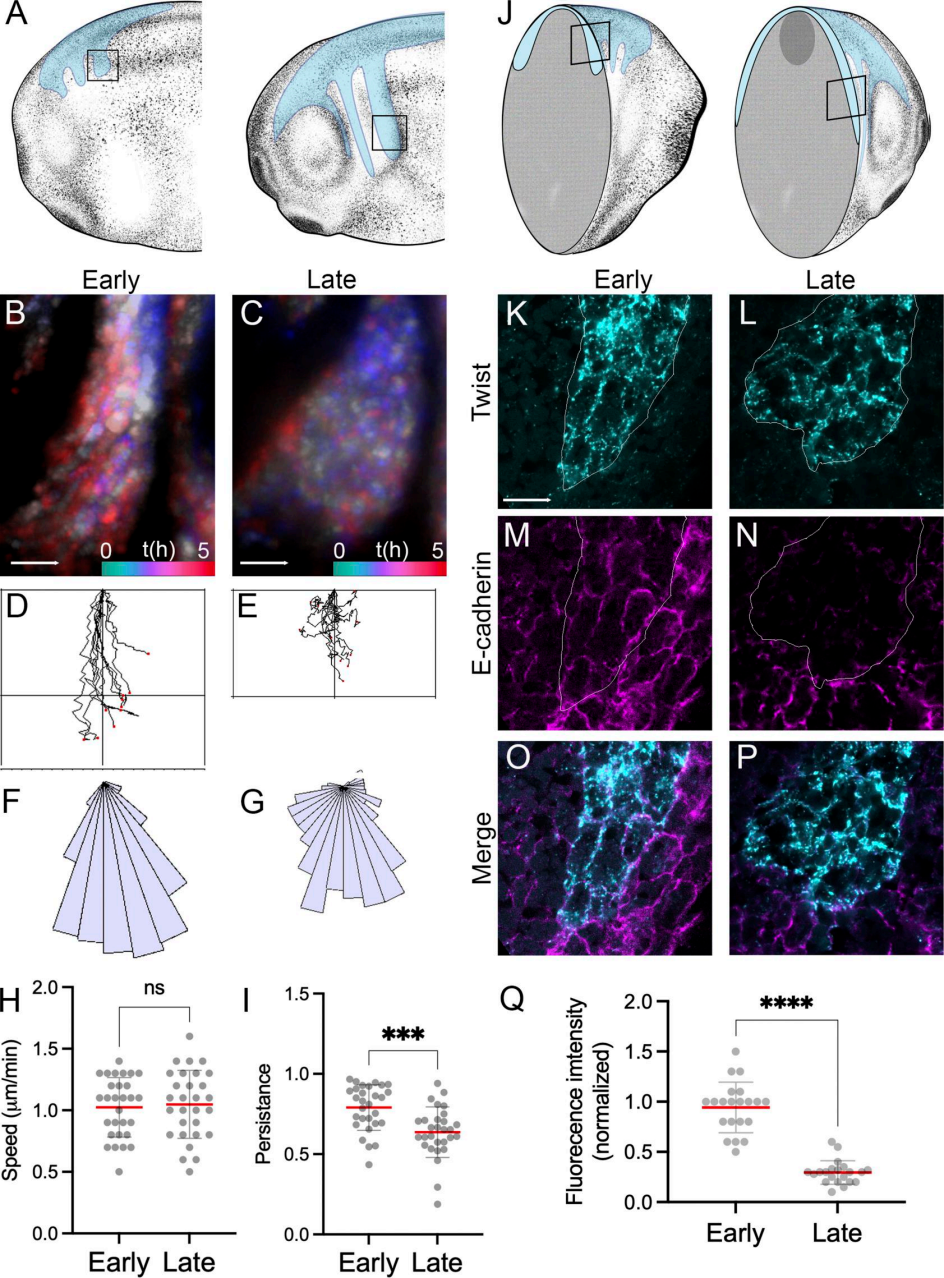
**E-cadherin is expressed in early migrating NC cells and reduced in late stream regions. (A)** Schematic showing the experimental approach where *X. laevis* embryos were injected at the eight-cell stage with nuclear marker (H2B-RFP), and NC migration was analyzed between stages 13–16 (early) and 17–25 (late). **(B and C)** Time-lapse imaging was performed on embryos in which the NC was labeled with nuclear-GFP. Frames of 5-h movies were color-coded and projected at early (B) and late (C) migratory stages. **(D and E)** Tracks of NC at the early (D) or late (E) migratory stages. **(F and G)** Angular distribution of migration direction in early and late stream regions. **(H and I)** Quantification of nuclear speed (H) and persistence (I) reveals reduced directional persistence at late stages. **(J)** Transverse cryosections of early and late migratory streams. **(K–Q)** E-cadherin immunostaining combined with twist mRNA detection by HCR in early (K, M, and O) and late (L, N, and P) NC cells shows progressive downregulation of E-cadherin in the NC stream regions (Q). Quantification of E-cadherin fluorescence intensity in early and late stream regions. Images are representative of at least three independent experiments. Scale bars in B, C: 50 µm; K: 10 µm. Error bars indicate mean ± SEM. Statistical analysis was performed using unpaired two-tailed Student’s *t* test. ***P ≤ 0.001; ****P ≤ 0.0001; n.s., not significant.

To investigate whether NC cells can initiate chemotaxis while still exhibiting epithelial characteristics, we analyzed migratory streams in *Xenopus* embryos from the beginning of NC migration (stage 13–16, hereafter referred to as “Early”) until more advanced migration (stage 17–25, “Late”). Unlabeled embryos were grafted with NC taken from embryos injected at the eight-cell stage with a nuclear marker to label NC nuclei (Theveneau et al., 2013), and nuclear trajectories were tracked at representative early and late migratory stages (Fig. 1 A). Early migrating NC exhibited more aligned and directional nuclear trajectories (Fig. 1 B) compared with those in the late stream, which showed less coordinated movement (Fig. 1 C), as quantified by trajectory analysis (Fig. 1, D and E) and migration angles (Fig. 1, F and G). Despite similar nuclear velocities (Fig. 1 H), persistence was significantly lower in late stream cells (Fig. 1 I), indicating less coordinated migration. As it is known that during *Xenopus* cephalic NC migration, cells undergo EMT, which is characterized by downregulation of E-cadherin (Barriga et al., 2013), we analyzed whether these behavioral changes (Fig. 1, A–I) were associated with differences in E-cadherin expression. We performed transverse cryosections (Fig. 1 J) at the equivalent stages described in Fig. 1, A–I and detected E-cadherin protein by immunostaining and twist mRNA by hybridization chain reaction (HCR), a classical NC marker. Early migrating NC cells exhibited high E-cadherin expression (Fig 1, K, M, and O), while late stream cells showed reduced levels (Fig 1, L, N, and P). These results suggest that NC cells can begin migrating while still retaining epithelial characteristics and that EMT may occur progressively along the migratory stream.

### NC clusters retain chemotactic ability despite increased E-cadherin expression

**Figure S1.**
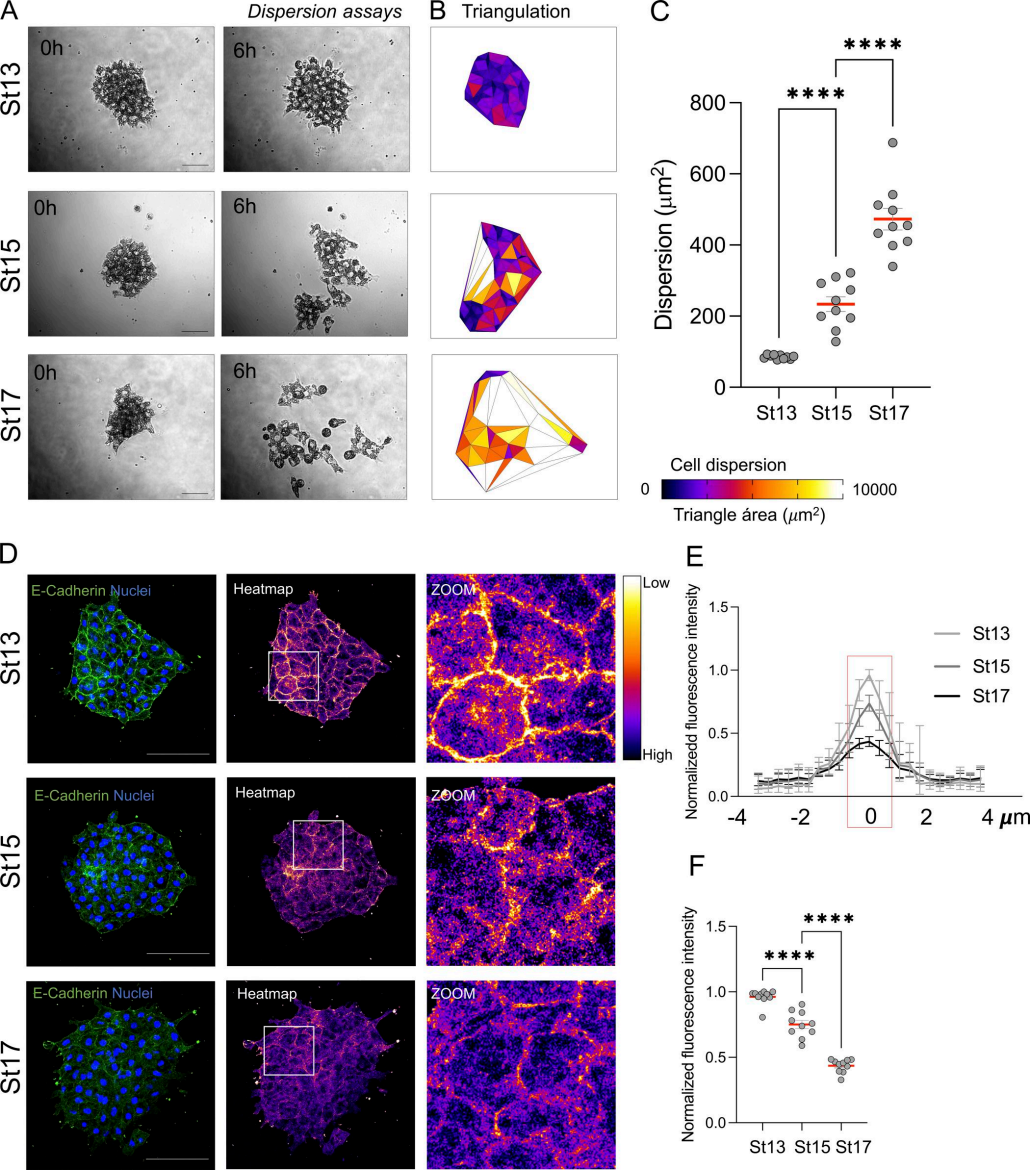
**Progressive changes in cell dispersion and E-cadherin levels across developmental stages of NC explants. (A)** Representative frames from time-lapse recordings of NC clusters dissected from St13, Stage 15, and St17. Clusters were plated on fibronectin and imaged over 6 h. Shown are the initial (0 h) and final (6 h) frames. Scale bar: 50 µm. **(B)** Visualization of dispersion at 6 h based on Delaunay triangulation of nearest-neighbor nuclei. Triangles are color-coded according to area. **(C)** Quantification of total dispersion area per cluster (*n* = 10 clusters per condition). **(D)** Representative immunofluorescence images showing E-cadherin localization in St13, Stage 15, and St17 NC clusters. Heatmaps indicate relative fluorescence intensity for visualization. Insets show magnified views of junctional regions extracted from the heatmap images. Scale bar: 100 µm. **(E)** Fluorescence intensity profiles along cell–cell junctions for NC clusters at St13, Stage 15, and St17. **(F)** Quantification of maximum junctional fluorescence intensity values (*n* = 10 clusters per condition). Each dot represents one cluster. Data are from at least three independent experiments. Error bars indicate mean ± SEM. Statistical analysis was performed using one-way ANOVA with Tukey’s multiple comparisons posttest. ****P ≤ 0.0001.

EMT is currently viewed as a gradual and reversible process rather than a discrete switch (Thompson et al., 2025; Yang et al., 2021). In this context, the classical view has been that NC cells reduce E-cadherin before migrating collectively. This downregulation acts as a signal that promotes increased motility and faster migration (Scarpa et al., 2015; Theveneau and Mayor, 2012; Barriga et al., 2013). However, our previous results (see Fig. 1) and other recent studies in various types of migrating cells that undergo EMT, such as cancer cells (Jolly et al., 2018; Shamir and Ewald, 2015), suggest that cells can retain high levels of E-cadherin and still migrate collectively. However, whether these epithelial clusters can undergo collective chemotaxis remains unknown. The *Xenopus* NC provides an ideal model to study this process, as both cell types, epithelial and mesenchymal, can be found migrating within the same stream during embryonic development. Although defining epithelial and mesenchymal organization in cells is not straightforward and remains debated, we followed the view in the literature that emphasizes behavioral rather than molecular criteria to describe these states (Yang et al., 2021). This behavioral framework is particularly relevant for NC cells, which undergo an EMT transition during development and progressively shift from cohesive to more dispersed migratory behaviors (Theveneau and Mayor, 2012; Scarpa et al., 2015; Shellard and Mayor, 2019). Accordingly, we refer to NC clusters as epithelial or mesenchymal based on their collective behavior. Epithelial clusters remain cohesive and maintain stable cell–cell contacts during migration, whereas mesenchymal clusters progressively lose cohesion, acquire front-rear polarity, and display greater dispersion over time. These modes represent dynamic states within a continuum of collective organization that the NC can adopt during development. To characterize these behaviors in the NC, we first examined how cluster organization changes across developmental stages. We analyzed NC clusters dissected from embryos at stages 13, 15, and 17, quantifying both their degree of dispersion and E-cadherin expression (Fig. S1 and Video 1). These analyses revealed a gradual decrease in E-cadherin levels accompanied by increased cell dispersion. Stage 13 (St13) clusters remained cohesive with high E-cadherin, stage 15 displayed moderate dispersion and intermediate levels of E-cadherin, and stage 17 (St17) showed greater dispersion and lower E-cadherin expression. Together, these results demonstrate a progressive shift from cohesive epithelial to dispersed mesenchymal organization as NC development proceeds.

**Video 1.** Shows the gradual transition in cell dispersion across St13, Stage 15, and St17 NC explants.

**Figure S2.**
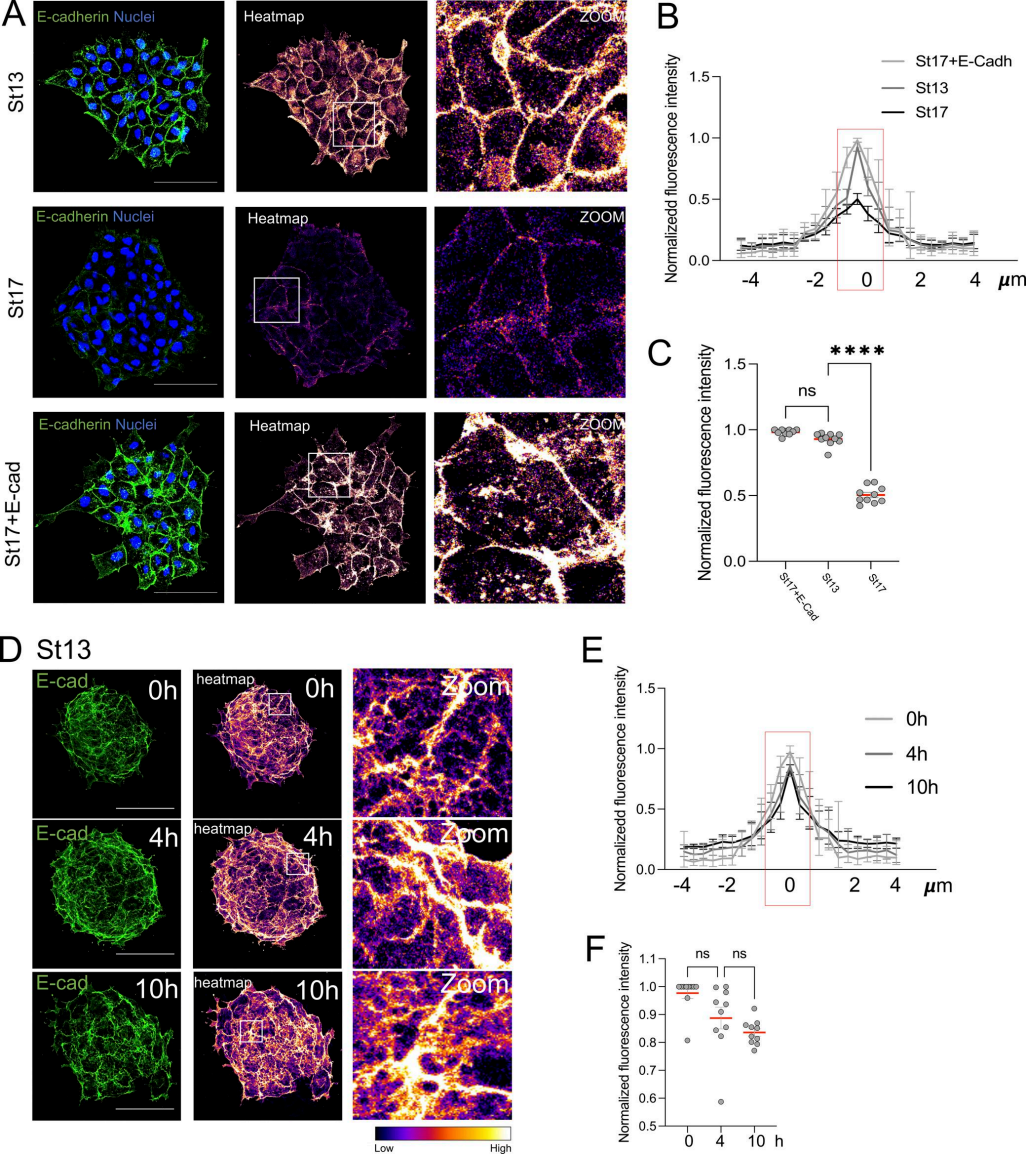
**E-cadherin levels in St13, St17, and St17 + E-Cad NC clusters, and stability of junctional E-cadherin during the chemotactic window in St13 explants. (A)** Immunofluorescence images of NC clusters from St13, St17, and St17 + E-Cad NC cells showing junctional E-cadherin distribution. Heatmaps show relative fluorescence intensity, and insets present enlarged views of selected junctions. Scale bar: 100 µm. **(B)** Fluorescence intensity profiles measured along cell–cell contacts for the conditions shown in A. **(C)** Quantification of peak junctional fluorescence intensity (*n* = 10 clusters per condition). **(D)** Immunofluorescence images of St13 NC clusters fixed at 0, 4, and 10 h after plating, illustrating E-cadherin localization over time. Heatmaps show relative intensity, with insets highlighting junctional regions. Scale bar: 100 µm. **(E)** Fluorescence intensity profiles along junctions at 0, 4, and 10 h. **(F)** Quantification of peak junctional fluorescence intensity shows stable E-cadherin levels throughout the 10-h interval (*n* = 10 clusters per condition). Each dot represents one cluster. Data are from at least three independent experiments. Error bars indicate mean ± SEM. Statistical analysis was performed using one-way ANOVA with Tukey’s multiple comparisons posttest. ****P ≤ 0.0001.

**Figure 2.**
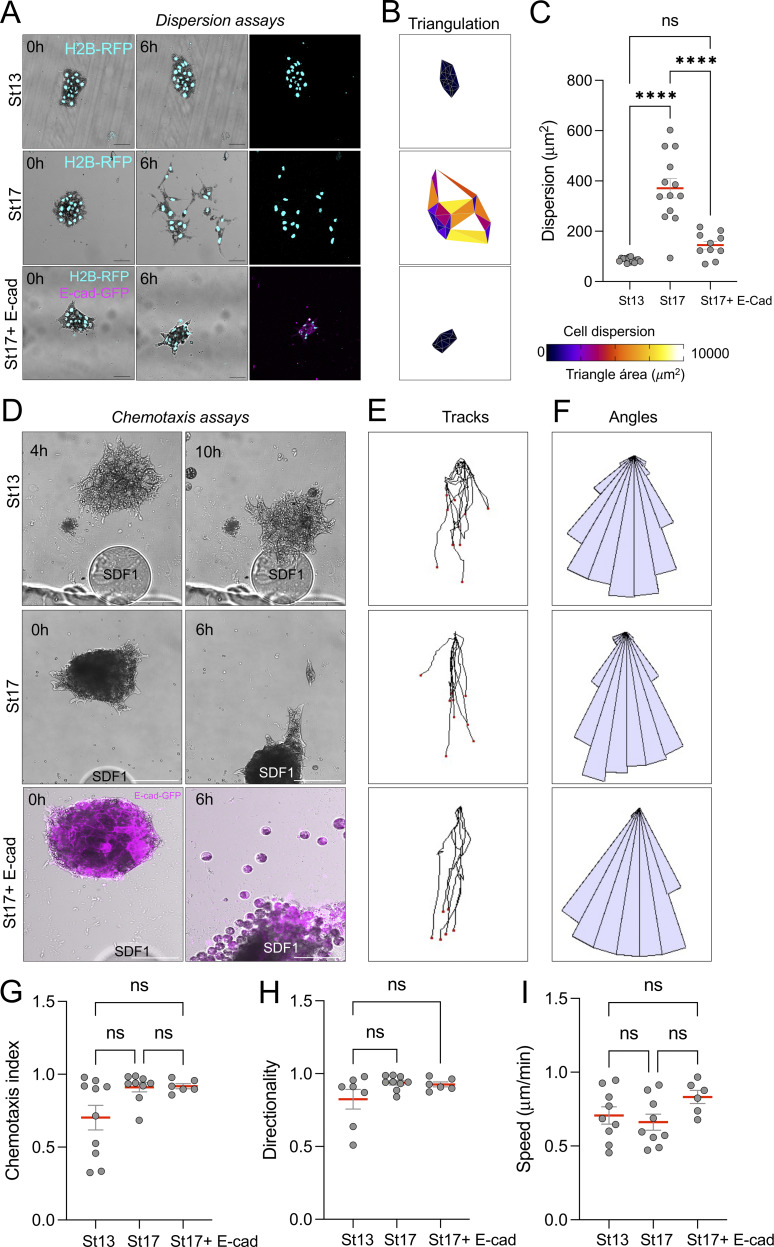
**E-cadherin inhibits cell dispersion but not chemotactic response in NC cells. (A)** Representative frames from time-lapse recordings of NC clusters dissected from *X. laevis* embryos at St13, St17, or St17 + E-Cad. The nucleus of NC was labeled with H2B-RFP, and NC clusters were plated on fibronectin and imaged over a 6-h period. Shown are the initial (0 h) and final (6 h) frames. Scale bar: 50 µm. **(B)** Visualization of dispersion based on the area between neighboring nuclei at 6 h. Triangles are color-coded according to size. **(C)** Quantification of total dispersion area per cluster. *n* = 14 (St13), 13 (St17), and 10 (St17 + E-Cad). **(D)** Representative images of NC clusters from each condition exposed to heparin beads soaked in purified SDF1. Scale bar: 100 µm. **(E)** Clusters centroid tracks over time relative to the position of the SDF1 source. **(F)** Angular distribution of cluster movement directions, represented as rose plots. **(G–I)** Quantification of chemotaxis index (G), directionality (H), and speed (I) of cluster centroid movement during time-lapse recordings. Data are representative of at least three independent experiments. Error bars indicate mean ± SEM. Statistical analysis was performed using one-way ANOVA with Tukey’s multiple comparisons posttest. ****P ≤ 0.0001; n.s., not significant.

As we aimed to compare epithelial versus mesenchymal chemotaxis, the comparison between St13 (epithelial) versus St17 (mesenchymal) NC is not ideal, because other developmental cofounding factors can affect cell behavior. To solve this issue, we aimed to compare the NC of the same developmental stage, but with different mesenchymal or epithelial behavior. As there is a strong correlation between these behaviors and the level of E-cadherin, we reasoned that we could induce epithelial conduct in St17 NC by overexpressing E-cadherin. To test this idea, we compared NC clusters dissected from three conditions: St13, St17, and St17 clusters overexpressing E-cadherin-GFP (St17 + E-Cad). In all cases, embryos were injected with H2B-RFP to label nuclei, and clusters were plated on fibronectin and imaged over 6 h. Before analyzing cluster behaviors, we quantified E-cadherin levels across these conditions. Immunofluorescence analysis showed that St17 + E-Cad clusters display E-cadherin levels comparable with epithelial St13 clusters and significantly higher than St17 controls (Fig. S2, A–C). These results indicate that E-cadherin overexpression restores epithelial levels without exceeding endogenous early NC levels. Next, we analyzed cell behavior under these three conditions. As expected, St17 clusters dispersed over time, showing the typical sign of mesenchymal behavior observed on migratory NC cells in the absence of external cues. In contrast, St13 and St17-E-cadherin–expressing clusters remained compact and showed no significant dispersion (Fig. 2, A–C and Video 2). These results confirm that E-cadherin is sufficient to prevent cluster dispersion in the absence of chemotactic signals, retaining its epithelial feature.

**Video 2.** Compares the cell dispersion of St13, St17, and St17 + E-cadherin clusters plated on fibronectin.

We then asked whether epithelial-like clusters (St13 and St17 + E-Cad) with high levels of E-cadherin could still respond to a chemotactic cue. To test this, we performed the SDF1 chemotaxis assays normally used for mesenchymal NC cells (Theveneau and Mayor, 2011; Theveneau et al., 2010). Surprisingly, all conditions were able to migrate toward the SDF1 source (Fig. 2, D–F and Video 3). It is important to note that St13 clusters began to migrate only after 4 h, showing a delay compared with St17 and E-cadherin overexpressing clusters. To confirm that St13 clusters maintained high E-cadherin levels during the migration period, we quantified expression at 4 and 10 h. St13 clusters retained high E-cadherin levels throughout this window, indicating that directed migration occurred while clusters preserved an epithelial-like state (Fig. S2, D–F). We then tracked the clusters in all three conditions from the moment they started migrating. Trajectory analysis showed no significant differences in chemotaxis index, directionality, or velocity between the three cluster conditions (Fig. 2, G–I). These results show that epithelial and mesenchymal NC can undergo similar collective chemotaxis toward SDF1.

**Video 3.** Shows chemotactic responses of NC clusters exposed to SDF1.

### E-cadherin alters the directional organization of cell movements within NC clusters during chemotaxis

Since St17 + E-Cad behave similarly to epithelial St13 clusters in terms of cohesion and chemotactic response (see Fig. 2), we decided to focus our subsequent analyses on St17–derived clusters. This approach allowed for more direct comparisons between clusters dissected at the same developmental stage, minimizing variability due to different developmental stages. From this point forward, we refer to St17 clusters as “mesenchymal” and to St17 + E-Cad-GFP as “epithelial-like,” consistent with the behavioral definitions established above.

**Figure 3.**
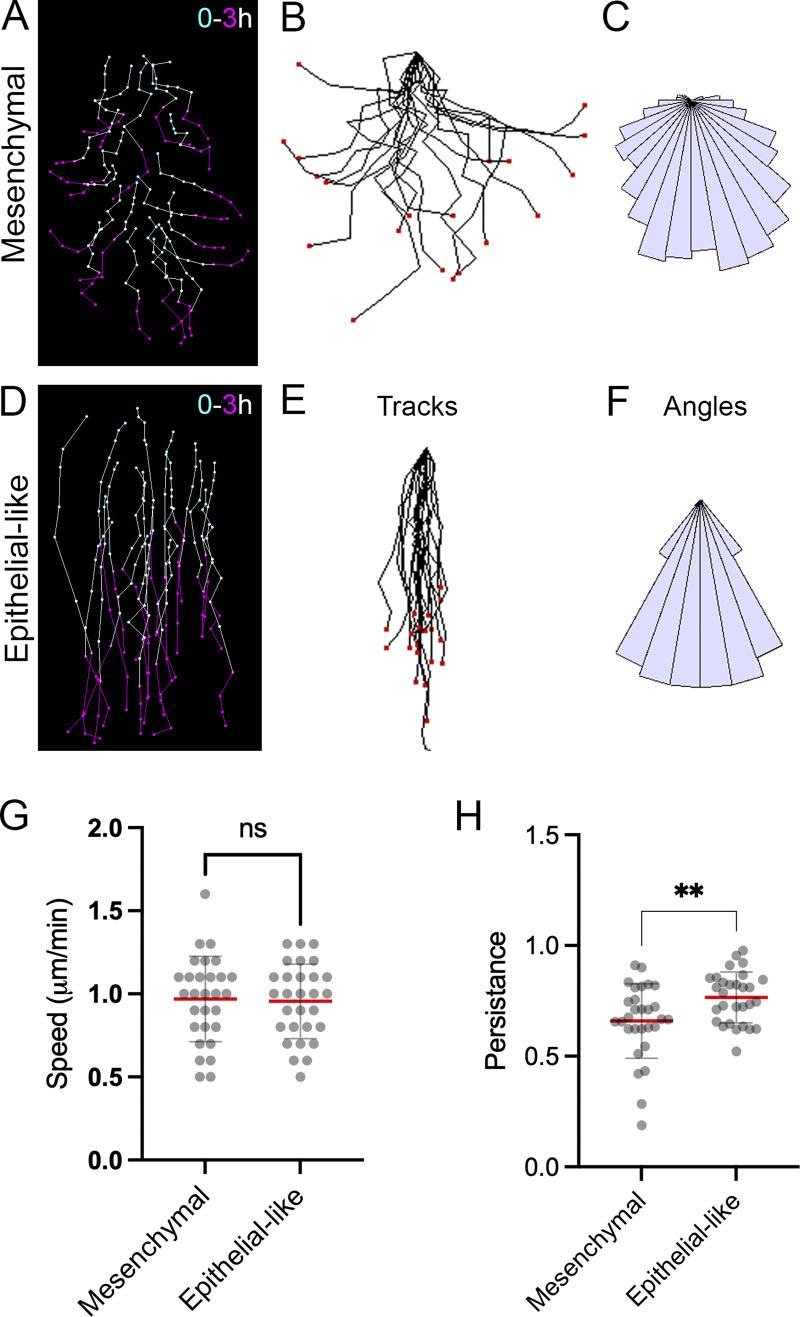
**E-cadherin alters the directional organization of cell movements within NC clusters during chemotaxis toward SDF1. (A and B)** Time color-coded images of nuclear trajectories in epithelial-like (A) and mesenchymal (B) NC clusters undergoing chemotaxis toward SDF1. **(C and D)** Tracking of nuclear positions from A and B. Scale bar: 50 µm. **(E and F)** Rose plots showing the angular distribution of nuclear movements from C and D, aligned to the main axis of cluster migration. **(G and H)** Quantification of nuclear speed (G) and persistence (H) based on nuclear tracks. Data are representative of at least three independent experiments. Error bars indicate mean ± SEM. Statistical analysis was performed using unpaired two-tailed Student’s *t* test; **P ≤ 0.01; n.s., not significant.

Although chemotaxis parameters such as chemotaxis index, directionality, and speed were not significantly different between conditions, we noticed that the migratory trajectories appeared more uniform in epithelial-like clusters compared with mesenchymal (Fig. 2, E and F). This observation led us to analyze the internal dynamics of individual nuclei to better understand the distinct mechanisms of migration operating within each cluster type during SDF1 chemotaxis. To this end, we tracked nuclear positions over time. Time color-coded trajectories revealed that nuclei in epithelial-like clusters moved in a highly directional and coordinated fashion, whereas mesenchymal clusters exhibited more disorganized nuclear paths (Fig. 3, A, B, D, and E). Angular distribution plots confirmed that epithelial-like nuclei were predominantly oriented along the axis of collective chemotaxis migration, while mesenchymal clusters exhibited a broader, less polarized pattern (Fig. 3, C and F). Quantification of nuclear velocity showed no significant difference between conditions (Fig. 3 G), but persistence was significantly reduced in mesenchymal clusters compared with epithelial-like clusters (Fig. 3 H). It is interesting to note that a similar difference in the coordinated migration of NC was observed *in vivo* when early (high E-cadherin) and late (low E-cadherin) stages were compared (Fig. 1). These data suggest that E-cadherin mediated cell–cell adhesion enhances the internal coordination of movement within migrating clusters by promoting cell alignment and directional persistence during SDF1 chemotaxis.

### Epithelial-like NC clusters lack supracellular actomyosin cables and accumulate pMLC at E-cadherin–mediated cell–cell junctions during chemotaxis

**Figure 4.**
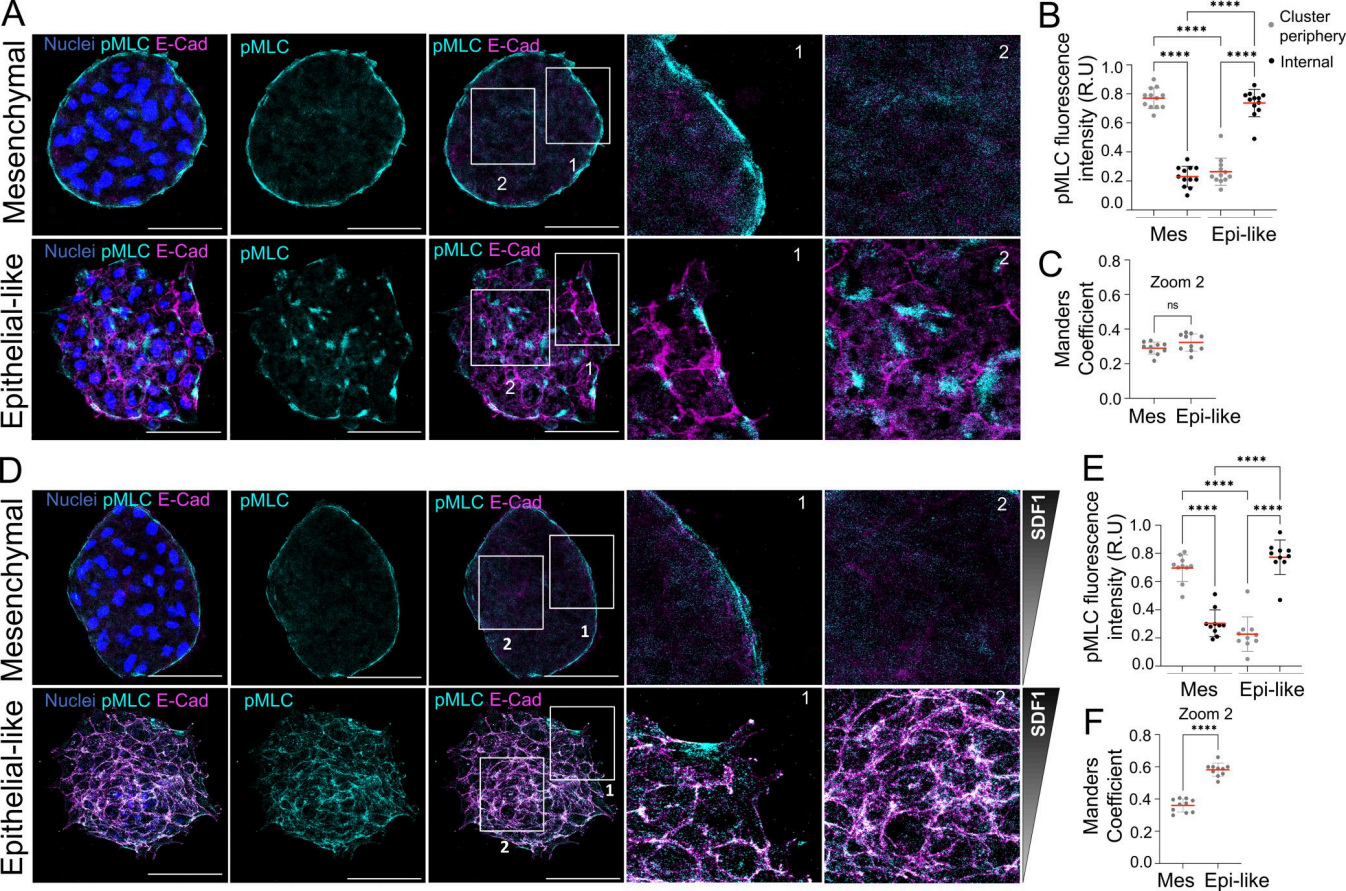
**E-cadherin impairs actomyosin cable formation and promotes pMLC accumulation at E-cadherin–mediated cell–cell contacts during SDF1 chemotaxis. (A)** Immunofluorescence staining of mesenchymal and epithelial-like NC clusters in the absence of external chemotactic cues, labeled for pMLC, E-cadherin, and nuclei. Zoom 1 shows the cluster periphery; Zoom 2 shows the central region of the same cluster. **(B)** Quantification of pMLC fluorescence intensity in the periphery and center of NC clusters shown in A (*n* = 12 clusters). **(C)** Quantification of colocalization between pMLC and E-cadherin using Mander’s coefficient in clusters without external cues (*n* = 10 clusters). **(D)** Immunofluorescence staining of mesenchymal and epithelial-like NC clusters during chemotaxis toward SDF1, labeled for pMLC, E-cadherin, and nuclei. Zoom 1 shows the cluster periphery; Zoom 2 shows the central region of the same cluster. Scale bar: 50 µm. **(E)** Quantification of pMLC fluorescence intensity in the periphery and center of clusters shown in D (*n* = 10 clusters). **(F)** Quantification of colocalization between pMLC and E-cadherin during SDF1 chemotaxis using Mander’s coefficient (*n* = 10 clusters). Colocalization is significantly higher in epithelial-like clusters. Each dot represents one cluster. Data are representative of at least three independent experiments. Error bars show mean ± SEM. Each dot represents one cluster. Statistical analysis was performed using one-way ANOVA with Tukey’s multiple comparisons posttest. ****P ≤ 0.0001; n.s., not significant.

**Figure S3.**
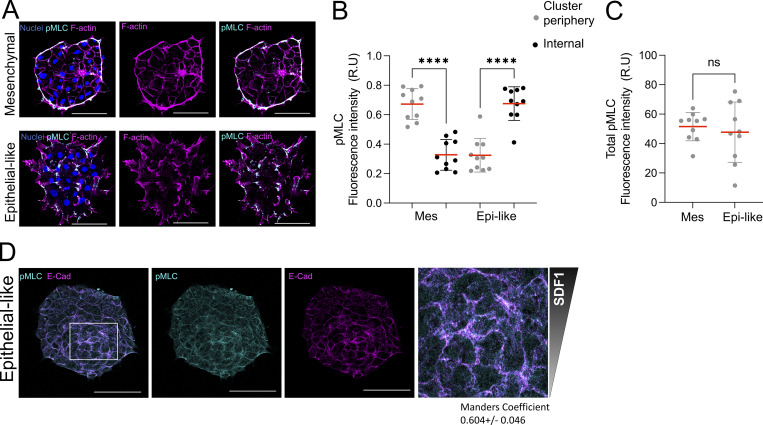
**E-cadherin disrupts supracellular actomyosin cable formation and redistributes pMLC toward E-cadherin–mediated junctions during SDF1 chemotaxis. (A)** Immunofluorescence staining of mesenchymal and epithelial-like NC clusters labeled for pMLC, F-actin, and nuclei. Mesenchymal clusters show a prominent peripheral actomyosin cable, whereas epithelial-like clusters lack this organization and instead exhibit pMLC and F-actin signal predominantly in internal regions, consistent with the presence of cryptic protrusions. Scale bar: 100 µm. **(B)** Quantification of pMLC fluorescence intensity in peripheral and central regions of the clusters shown in A (*n* = 10 clusters). Each dot represents one cluster. Statistical analysis was performed using one-way ANOVA with Tukey’s multiple comparisons posttest. ****P ≤ 0.0001; n.s., not significant. **(C)** Quantification of total pMLC fluorescence intensity of NC clusters shown in Fig. 4 (*n* = 12 clusters). Each dot represents one cluster. Statistical analysis was performed using an unpaired two-tailed Student’s *t* test. ****P ≤ 0.0001; n.s., not significant. **(D)** Immunofluorescence staining of epithelial-like NC clusters during chemotaxis toward SDF1, showing E-cadherin and pMLC at the basal z-plane. A single optical section reveals colocalization between E-cadherin and pMLC at substrate-associated junctions, suggesting E-cadherin–dependent pMLC recruitment to cryptic protrusions. Zoom shows a central region of the same cluster. Data are representative of at least three independent experiments. Scale bar: 50 µm. Error bars show mean ± SEM.

Previous work demonstrated that mesenchymal NC clusters form a supracellular actomyosin cable at their periphery, which is essential for driving collective chemotaxis (Shellard and Mayor, 2019; Shellard et al., 2018). However, it remains unknown how epithelial-like clusters, which have high levels of E-cadherin, organize their actomyosin network during SDF1 chemotaxis. To explore this, we performed immunofluorescence staining for phosphorylated myosin light chain (pMLC) in mesenchymal and epithelial-like clusters, both in the absence of chemotactic cues and during chemotaxis toward SDF1. Without any external cue, mesenchymal clusters displayed the expected peripheral enrichment of pMLC, consistent with the formation of a contractile actomyosin cable (Fig. 4 A, mesenchymal zoom 1). This organization was confirmed by phalloidin staining acquired together with pMLC (Fig. S3, A and B), which showed F-actin colocalizing with pMLC along the cluster edge, consistent with a peripheral actomyosin cable. In contrast, epithelial-like clusters lacked this peripheral organization and instead showed increased pMLC signal at the center of the cluster, particularly in regions adjacent to E-cadherin–mediated cell–cell junctions (Fig. 4 A, epithelial-like zoom 2), with F-actin and pMLC accumulated internally in cryptic protrusions, rather than forming a peripheral actomyosin cable (Fig. S3, A and B). Quantification confirmed significantly lower pMLC intensity at the periphery compared with center in epithelial-like clusters (Fig. 4 B). Mander’s coefficient analysis revealed no significant differences in colocalization between pMLC and E-cadherin in between mesenchymal and epithelial-like clusters measured in internal region of the clusters in the absence of external cues (Fig. 4 C). Interestingly, during chemotaxis toward SDF1, mesenchymal clusters maintained their peripheral actomyosin cable (Fig. 4 D, mesenchymal Zoom 1), while epithelial-like clusters continued to accumulate pMLC in the internal regions, particularly at cell–cell junctions where E-cadherin appears to stabilize pMLC (Fig. 4 D, epithelial-like zoom 2). This redistribution was confirmed by fluorescence intensity quantification (Fig. 4 E), and colocalization analysis using Mander’s coefficient revealed a nearly twofold increase in the colocalization between pMLC and E-cadherin in epithelial-like clusters during chemotaxis compared with the condition without external cues (Fig. 4 F). Consistent with this, quantification of total pMLC levels during chemotaxis showed no significant differences between mesenchymal and epithelial-like clusters (Fig. S3 C), indicating that chemotaxis primarily alters the spatial distribution of pMLC rather than changing the total amount of pMLC. To further confirm that pMLC is associated with E-cadherin at cryptic protrusions, we analyzed a single focal plane at basal z-level, where these protrusions are typically observed and detected a prominent overlap between both signals at this level (Fig. S3 D). These results indicate that epithelial-like clusters, unlike mesenchymal, do not form a peripheral actomyosin cable but instead accumulate pMLC at cell–cell junctions mediated by E-cadherin during chemotaxis. This shows that the mode of actomyosin organization is fundamentally different between the two collective modes of migration during SDF1 chemotaxis.

### Protrusion dynamics and polarity differ between mesenchymal and epithelial-like clusters during chemotaxis

**Figure 5.**
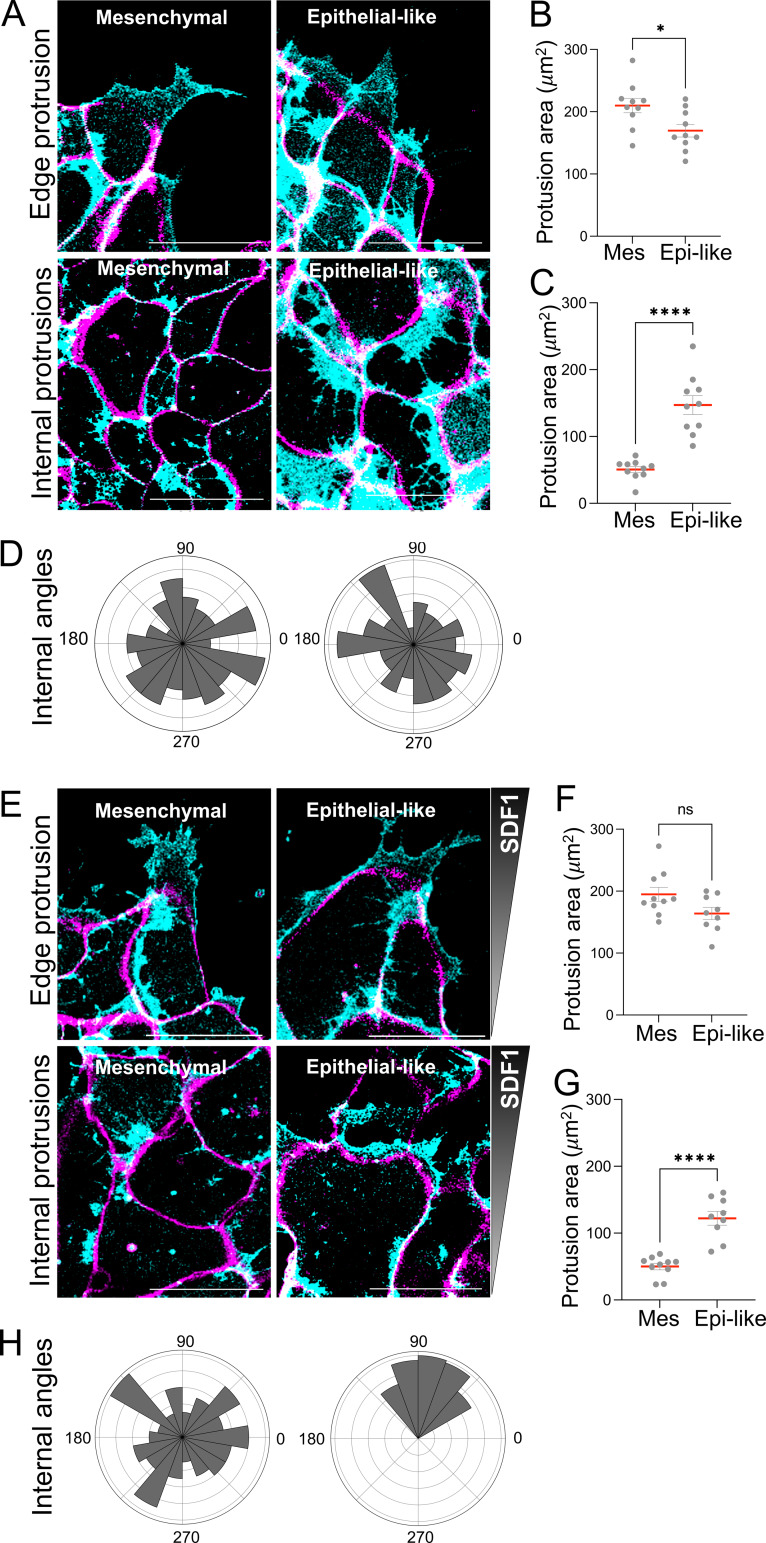
**SDF1 chemotaxis differentially regulates protrusion dynamics and polarization in epithelial-like and mesenchymal NC clusters. (A)** Representative images of mesenchymal and epithelial-like NC clusters without chemotactic cues. Cells were labeled with mbmCherry. Optical sections were acquired at the substrate level (pseudocolored in cyan, showing protrusions) and above the substrate (pseudocolored in magenta to highlight cell bodies) and overlaid to distinguish both planes. **(B and C)** Quantification of protrusion area at the cluster edge (mesenchymal, *n* = 10; epithelial-like, *n* = 9) and (C) internally cryptic protrusion (*n* = 10 per condition) in the absence of external chemotactic cues. **(D)** Orientation of internal protrusions measured as angles in mesenchymal and epithelial-like clusters (*n* = 10 per condition) without chemotactic cues. **(E)** Representative images of mesenchymal and epithelial-like NC clusters during chemotaxis to SDF1. **(F and G)** Quantification of protrusion area at the cluster edge (mesenchymal, *n* = 10; epithelial-like, *n* = 9) and (G) internally cryptic protrusion (mesenchymal, *n* = 10; epithelial-like, *n* = 9) during chemotaxis. **(H)** Orientation of internal protrusions during chemotaxis, showing polarization toward the SDF1 in epithelial-like clusters (*n* = 10 mesenchymal; *n* = 9 epithelial-like). Scale bar: 30 µm. Each dot represents one cluster. Data are from at least three independent experiments. Error bars show mean ± SEM. Statistical analysis was performed using unpaired two-tailed Student’s *t* test. ****P ≤ 0.0001; *P ≤ 0.05.

Protrusion dynamics are central to collective cell migration, as they define how clusters interact with their substrate and generate traction (Trepat and Sahai, 2018; Haeger et al., 2015; Friedl and Gilmour, 2009). To investigate how protrusions contribute to the distinct migratory behaviors of mesenchymal and epithelial-like clusters during chemotaxis, we used membrane-targeted mCherry (mbmCherry) to visualize protrusive activity at both the periphery and internal regions of clusters. Z-sections were acquired at the substrate level (highlighting protrusions; displayed in cyan) and above the substrate (highlighting cell bodies; displayed in magenta) and overlaid to distinguish both planes for quantification. In the absence of chemotactic cues, mesenchymal clusters seem to have slightly larger edge protrusions compared with epithelial-like clusters (Fig. 5, A and B). In contrast, epithelial-like clusters exhibited greater internal cryptic protrusions than mesenchymal clusters (Fig. 5, A–C and Video 4), a feature commonly associated with epithelial phenotypes. These internal cryptic protrusions appeared randomly oriented in both conditions (Fig. 5 D). During chemotaxis toward SDF1, edge protrusions in both cluster types remained similar in area (Fig. 5, E and F). Epithelial-like clusters continued to exhibit larger cryptic protrusions compared with mesenchymal (Fig. 5, E–G and Video 5). Importantly, these internal protrusions in epithelial-like clusters became strongly polarized toward the SDF1 source, as shown by the angular distribution analysis (Fig. 5 H). In contrast, mesenchymal clusters still exhibited non-polarized internal protrusions under the same conditions. These findings suggest that epithelial-like clusters directionally reorganize their internal protrusions during chemotaxis. Together, these data support the idea that, in addition to leading edge protrusions, internal cryptic protrusions contribute to guiding the collective migration of epithelial-like clusters toward SDF1, potentially through coordination with E-cadherin–mediated adhesion. Similar protrusions have been described in epithelial sheets and collectively migrating tissues (Farooqui and Fenteany, 2005; Reffay et al., 2014; Shamir and Ewald, 2015) and may help organize directional movement in response to chemotactic cues (Mayor and Etienne-Manneville, 2016).

**Video 4.** Shows internal cryptic protrusions in the absence of external cues.

**Video 5.** Shows polarization of internal protrusions in epithelial-like clusters during chemotaxis.

### Epithelial-like clusters polarize Rac1-GTP internally during chemotaxis, while mesenchymal clusters retain peripheral activation

**Figure 6.**
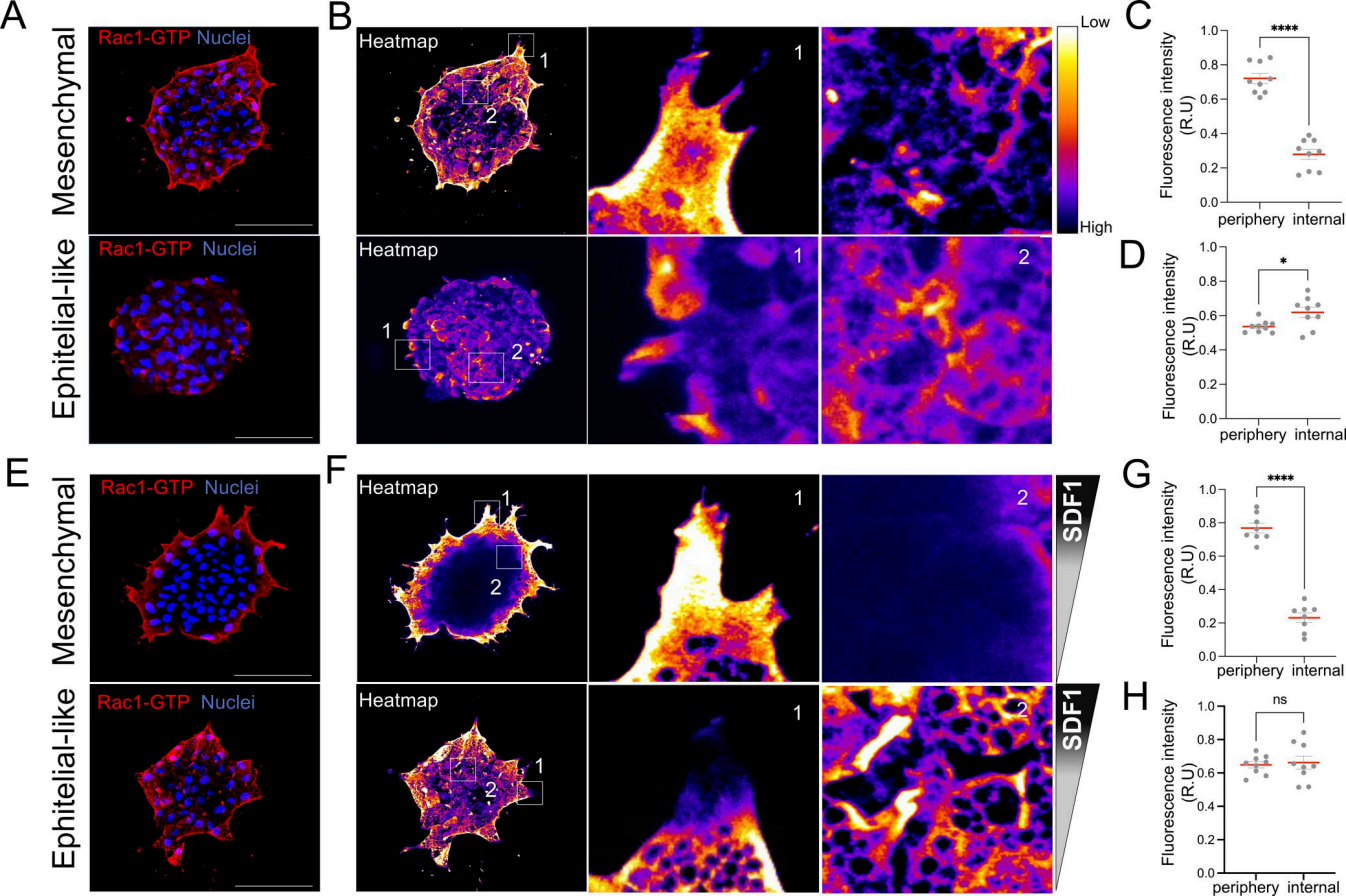
**Rac1-GTP localizes to the periphery in mesenchymal clusters and polarizes internally in epithelial-like clusters during SDF1 chemotaxis. (A)** Immunostaining of active Rac1 (Rac1-GTP) in mesenchymal and epithelial-like NC clusters. Scale bar: 100 µm. **(B)** Corresponding fluorescence heatmaps showing Rac1-GTP distribution. **(C and D)** Zoom 1 highlights the cluster periphery; Zoom 2 shows the internal region. Quantification of Rac1-GTP fluorescence intensity in mesenchymal (C) and epithelial-like (D) clusters in the absence of chemotactic cue, comparing peripheral and internal regions (*n* = 9 per region per condition). **(E)** Immunostaining of Rac1-GTP in mesenchymal and epithelial-like clusters during chemotaxis to SDF1. Scale bar: 100 µm. **(F)** Heatmaps of Rac1-GTP signal during chemotaxis. Zoom 1 and 2 show peripheral and internal cluster regions, respectively. **(G and H)** Quantification of Rac1-GTP fluorescence intensity in mesenchymal (G) (*n* = 8) and epithelial-like (H) (*n* = 9) clusters during chemotaxis. Each dot represents one cluster. Data are from at least three independent experiments. Error bars show mean ± SEM. Error bars show mean ± SEM. Statistical analysis was performed using unpaired two-tailed Student’s *t* test. ****P ≤ 0.0001; *P ≤ 0.05.

Actin-based protrusions are essential for directed migration and are tightly regulated by the small GTPase Rac1, which promotes actin polymerization in migrating cells (Matthews et al., 2008; Raftopoulou and Hall, 2004). During collective migration, Rac1 activation can be spatially polarized depending on cell–cell interactions and external cues (Rorth, 2012; Reffay et al., 2014). Previous studies have shown that Rac1 activity has been associated with polarized protrusion formation, particularly at the free edge of the mesenchymal NC cells (Scarpa et al., 2015). However, how Rac1 activity is spatially reorganized during chemotaxis in NC remains unknown. To address this, we analyzed the localization of active Rac1 (Rac1-GTP) in mesenchymal and epithelial-like NC clusters during SDF1 chemotaxis. In the absence of chemotactic cues, Rac1-GTP was predominantly enriched at the periphery of mesenchymal clusters, consistent with protrusive activity at the edge of clusters (Fig. 6, A–C, mesenchymal zoom 1). Epithelial-like clusters also showed Rac1 activity at the edges of the clusters, but Rac1GTP was also observed at the cluster centers (Fig. 6, A–D, epithelial-like zoom 2). During chemotaxis toward SDF1, mesenchymal clusters maintained high levels of Rac1-GTP at their periphery, with clear polarization at the leading-edge protrusions oriented toward the SDF1 gradient (Fig. 6, E–G, mesenchymal zoom 1), supporting the idea that free-edge protrusions guide directional migration. Although epithelial-like maintained Rac1-GTP at the leading edge protrusion, the cluster showed a tendency for Rac1GTP to accumulate within internal regions, often aligned with the direction of the SDF1 source (Fig. 6, E–H, epithelial-like zoom 2). These findings indicate that epithelial-like and mesenchymal clusters adopt different active Rac1 distribution patterns during chemotaxis. While mesenchymal clusters depend on peripheral Rac1 activity, epithelial-like clusters retain some Rac1 activity at the edge but also show enriched internal Rac1-GTP localization, consistent with reports that intercellular adhesion can influence Rac1 polarity in epithelial collectives (Yamada and Nelson, 2007), possibly enabling directional cryptical protrusion coordination through E-cadherin–mediated cell–cell contacts.

### Focal adhesions relocate from edge protrusions to internal regions of the cluster in epithelial-like clusters during chemotaxis

**Figure 7.**
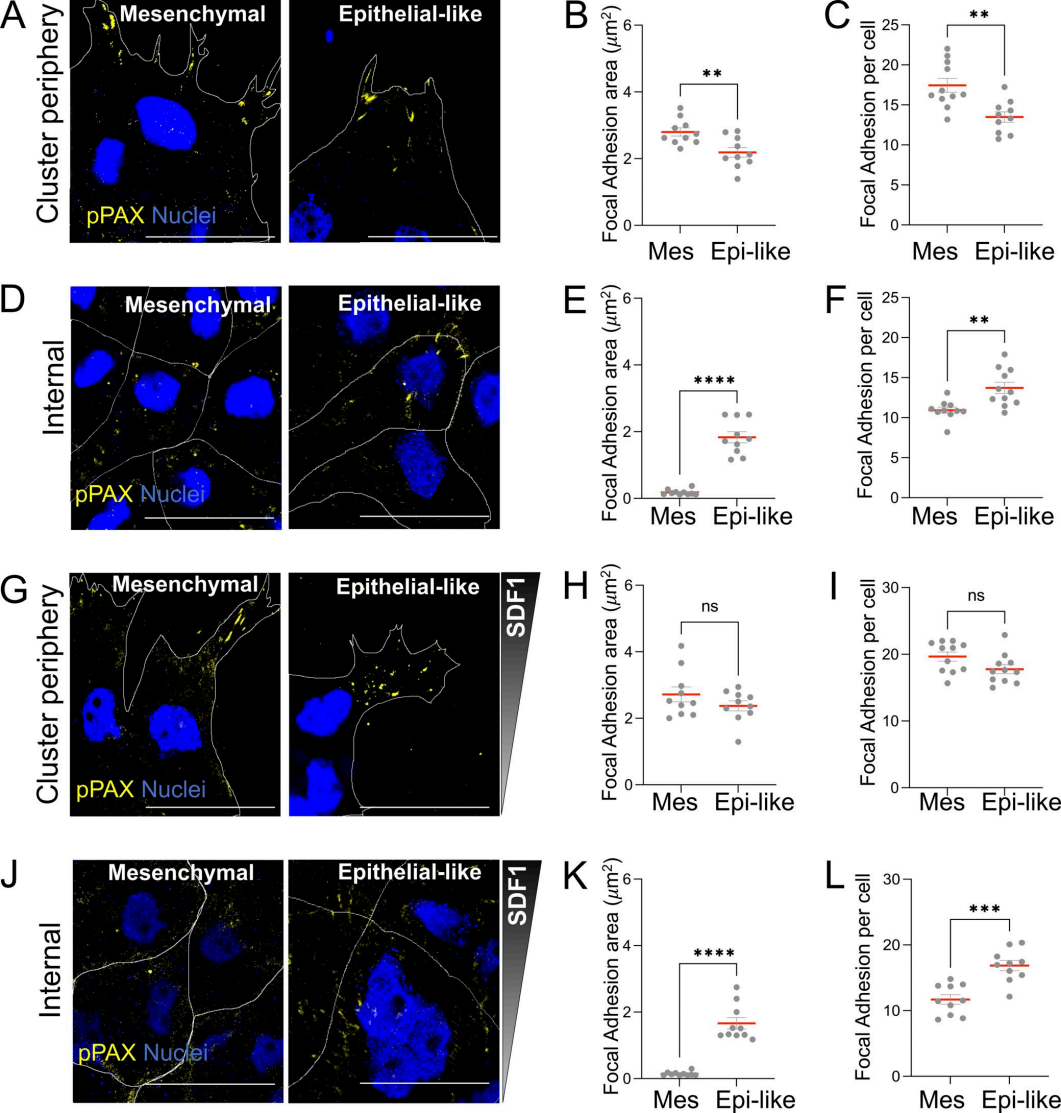
**FAs redistribute from protrusions in mesenchymal clusters to internal regions of the cluster in epithelial-like clusters during chemotaxis toward SDF1. (A)** Immunofluorescence staining of mesenchymal and epithelial-like NC clusters in the absence of external chemotactic cues, labeled for pPAX and nuclei. The region shown corresponds to edge protrusions. **(B and C)** Quantification of FA area and number at edge protrusions in NC clusters shown in A (*n* = 10 clusters per condition). **(D)** Immunofluorescence staining of mesenchymal and epithelial-like NC clusters in the absence of external chemotactic cues, labeled for pPAX and nuclei. The region shown is the inner cluster region. **(E and F)** Quantification of FA area and number in internal regions of the cluster in NC clusters shown in D (*n* = 10 clusters per condition). **(G)** Immunofluorescence staining of mesenchymal and epithelial-like NC clusters during chemotaxis toward SDF1, labeled for pPAX and nuclei. The region shown corresponds to edge protrusions. **(H and I)** Quantification of FA area and number at edge protrusions in NC clusters shown in G (*n* = 10 clusters per condition). **(J)** Immunofluorescence staining of mesenchymal and epithelial-like NC clusters during chemotaxis toward SDF1, labeled for pPAX and nuclei. The region shown corresponds to the cluster interior. **(K and L)** Quantification of FA area and number in the internal regions in NC clusters shown in J (*n* = 10 clusters per condition). Scale bars: 30 µm. Each dot represents one cluster. Data are from at least three independent experiments. Error bars show mean ± SEM. Error bars show mean ± SEM. Statistical analysis was performed using unpaired two-tailed Student’s *t* test. ****P ≤ 0.0001; ***P < 0.001; **P ≤ 0.01.

Since we observed that pMLC localization is observed in supracellular cables in mesenchymal clusters, while it remains in the E-cadherin–mediated junctions in epithelial-like clusters during chemotaxis (see Fig. 4), we next asked whether a similar distribution occurred in focal adhesion (FA) components. We focused on phospho-paxillin (p-PAX), which is recruited early during FA formation and has been associated with adhesion dynamics and force transmission (Deakin and Turner, 2008; Pasapera et al., 2010). Moreover, p-PAX organization has been shown to differ between epithelial and mesenchymal migration modes and to be influenced by E-cadherin–mediated junctions in collective migration (Mertz et al., 2013; Bazellières et al., 2015). In the absence of chemotactic cues, mesenchymal clusters exhibited a significant increase in both the number and area of p-PAX–positive FAs at edge protrusions when compared with epithelial-like clusters (Fig. 7, A–C). In contrast, epithelial-like clusters exhibited a greater number and area of p-PAX–positive adhesion in the inner cluster region (Fig. 7, D–F), indicating a basal redistribution of adhesion complexes toward internal junctional regions. Upon exposure to an SDF1 gradient, in mesenchymal clusters, FAs remained concentrated at edge protrusions and tended to be larger than in epithelial-like clusters, although the difference was not statistically significant (Fig. 7, G–I). In contrast, epithelial-like clusters showed a marked increase in number and total FA area within the internal regions of the cluster in comparison with mesenchymal clusters during chemotaxis (Fig. 7, J–L), further supporting a shift in adhesive force transmission from protrusive edges to internal regions. This redistribution parallels the pattern observed for pMLC and Rac1, suggesting coordinated reorganization of contractile and adhesive structures in epithelial-like clusters toward the internal regions of the cluster during SDF1 chemotaxis.

### Traction forces redistribute from edge protrusions in mesenchymal clusters to E-cadherin–mediated cell–cell junctions in epithelial-like clusters during chemotaxis

**Figure 8.**
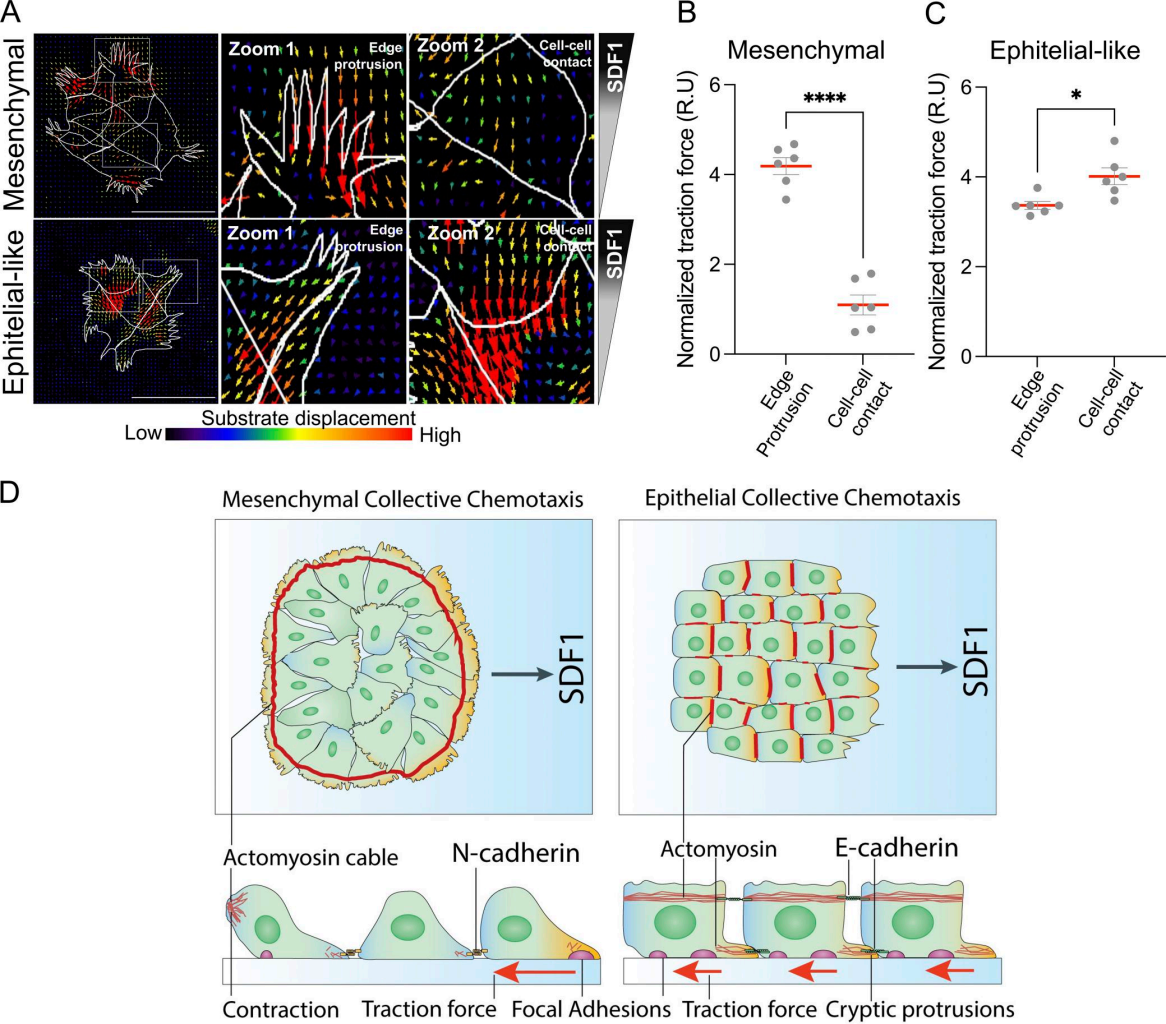
**Traction forces concentrate at edge protrusions in mesenchymal clusters and redistribute toward cell–cell contacts in epithelial-like clusters during SDF1 chemotaxis. (A)** TFM showing substrate displacement maps in mesenchymal and epithelial-like NC clusters during chemotaxis. Zooms highlight edge protrusions and cell–cell contact regions. **(B and C)** Quantification of normalized traction force at edge protrusions and cell–cell contact in NC clusters shown in mesenchymal and (C) epithelial-like (*n* = 6). Each dot represents one cluster. Data are from at least three independent experiments. Error bars show mean ± SEM. Error bars show mean ± SEM. Statistical analysis was performed using an unpaired two-tailed Student’s *t* test. ****P ≤ 0.0001; *P ≤ 0.05. Scale bars: 100 µm. **(D)** Model summarizing the distinct strategies of collective chemotaxis. In mesenchymal clusters, traction is generated primarily at the leading edge, driven by supracellular actomyosin cables and polarized protrusions, and cell–cell cohesion is maintained by N-cadherin–mediated adhesion. In epithelial-like clusters, traction redistributes toward junctional regions, where E-cadherin adhesion and actomyosin coupling coordinate collective movement. Orange represents Rac1 activity.

**Figure S4.**
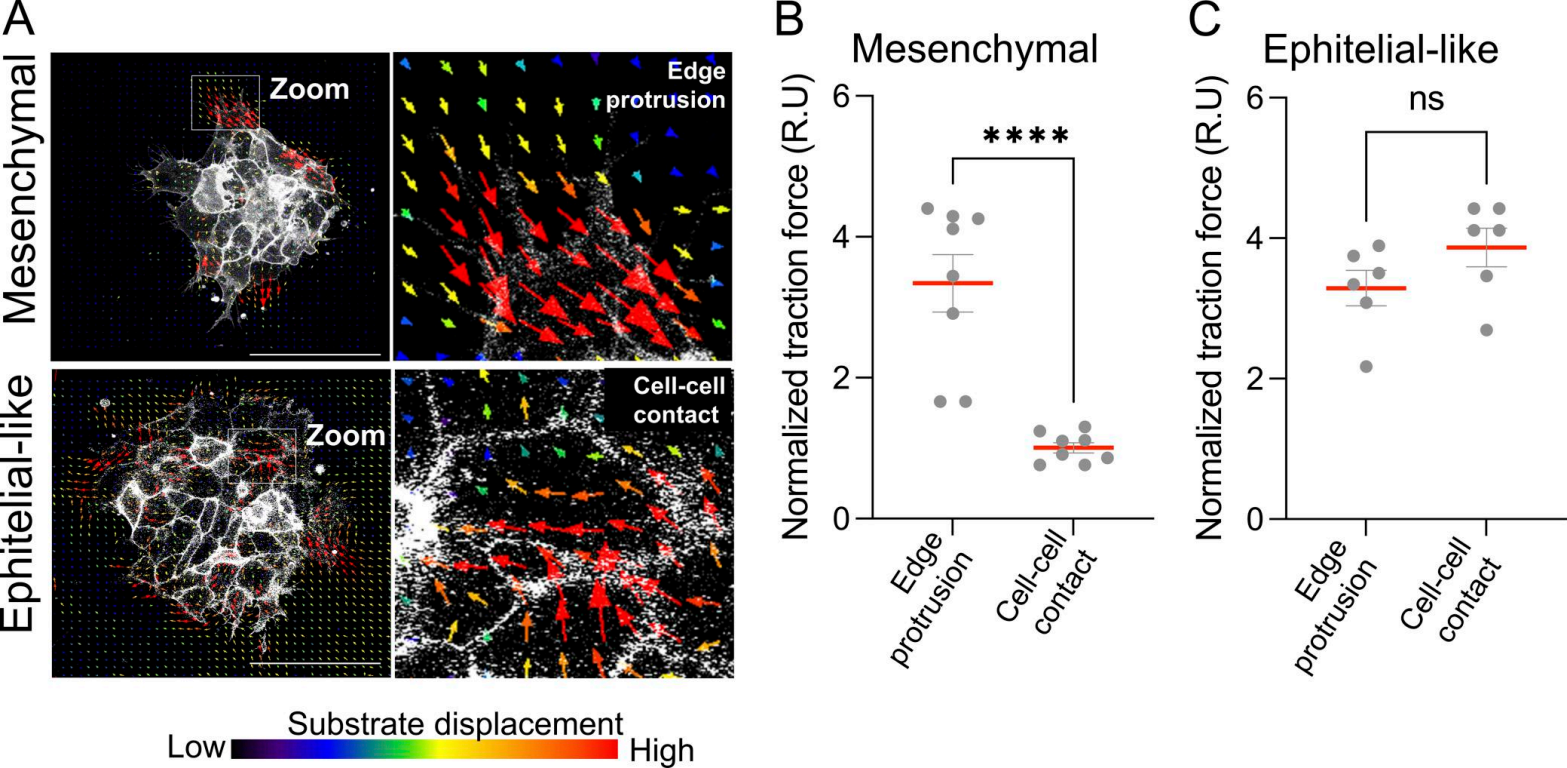
**Traction forces display distinct spatial distributions in the absence of chemotactic cues. (A)** TFM showing substrate displacement maps in mesenchymal and epithelial-like NC clusters in the absence of external chemotactic cues. Zooms highlight edge protrusions and cell–cell contact regions. **(B and C)** Quantification of normalized traction force at edge protrusions and cell-cell contact in NC clusters shown in mesenchymal and (C) epithelial-like. Data are from at least three independent experiments. Scale bars: 100 µm. Error bars show mean ± SEM. Statistical analysis was performed using an unpaired two-tailed Student’s *t* test. ****P ≤ 0.0001; ns, not significant.

To determine how the redistribution of mediators of contractility and adhesion components previously described impacts force transmission during chemotaxis, we performed TFM in mesenchymal and epithelial-like NC clusters, both in the absence and presence of SDF1 gradient. During SDF1-induced chemotaxis, mesenchymal clusters maintained high traction forces at the edge of the cluster, particularly at the leading front (Fig. 8 A, mesenchymal), correlating with edge protrusions and FA. However, epithelial-like clusters exhibited a marked shift in traction force localization, showing increased force at internal cell–cell junctions and reduced force at the edge compared with mesenchymal clusters (Fig. 8 A, epithelial-like). This redistribution of traction suggests that force transmission occurs through intercellular adhesions within the cluster, consistent with previous studies showing that coordinated migration can emerge from collective tension across junctions (Tambe et al., 2011). Quantification confirmed that chemotaxis was associated with an increase in traction forces at cell–cell contacts in epithelial-like clusters (Fig. 8 C), while mesenchymal clusters retained primarily peripheral force distribution (Fig. 8 B). A similar distribution of traction forces between central and peripheral clusters in the absence of a chemoattractant was observed (Fig. S4). Together, these results suggest that epithelial-like clusters tend to distribute traction forces at E-cadherin–stabilized cell–cell junctions during chemotaxis. This redistribution may reflect a distinct force transduction strategy that contributes to collective migration in the absence of supracellular cables. A similar mechanism of long-range force transmission has been proposed to drive collective durotaxis in epithelial sheets (Sunyer et al., 2016), suggesting that internal traction can support coordinated movement even without supracellular contractile structure.

## Discussion

Our results reveal an alternative mode of collective chemotaxis in NC cells, where epithelial-like organization is maintained throughout directed migration toward SDF1. This is reminiscent of observations in epithelial tissues, where E-cadherin–positive clusters migrate collectively and efficiently, despite maintaining strong cell–cell adhesion (Cheung et al., 2013; Balasubramaniam et al., 2021; Campàs et al., 2024; Gupta and Yap, 2021; Labernadie et al., 2017; Padmanaban et al., 2019; Sonam et al., 2023). While mesenchymal NC clusters use polarized edge protrusions, supracellular actomyosin cables, and rear contraction to drive directional movement (Shellard et al., 2018; Theveneau et al., 2010; Carmona-Fontaine et al., 2008; Shellard and Mayor, 2021). Epithelial-like clusters migrate efficiently through coordinated cell displacements (Fig. 3), internal polarized protrusions (Fig. 5), and a redistribution of Rac1 activity toward the cluster interior (Fig. 6). These observations suggest that Rac1 may promote actin polymerization at internal sites of protrusive activity, possibly regulated by cell–cell adhesion. Our results resonate with findings in epithelial monolayers, where cryptic lamellipodia arise adjacent to adherens junctions and are actively regulated by E-cadherin–dependent signaling (Ozawa et al., 2020). In particular, this study showed that adherens junctions serve not only as mechanical links but also as platforms to organize protrusive activity at internal cell–cell contacts. This internal coordination supports chemotactic guidance even in the absence of peripheral supracellular structures and highlights the mechanical plasticity of collective migration in NC cells. This spatial organization of Rac1 activity is consistent with the mechanism reported in mammary epithelial cells, where the MYO6-DOCK7 axis restricts Rac1 activation to specific internal sites, allowing the formation of polarized cryptic lamellipodia during collective migration (Menin et al., 2023). Our results build on this concept by showing that, in epithelial-like NC clusters, internal Rac1 often aligns with the chemotactic axis, suggesting that junction-associated Rac1 signaling can be repurposed to support directed migration in a developmental context. Although previous models of NC migration emphasize the role of supracellular contractile cables and leader-cell protrusions (Shellard et al., 2018; Theveneau et al., 2010), our findings show that these features are not the only ones required for efficient chemotaxis. For instance, epithelial-like clusters, which lack actomyosin cables under baseline conditions, maintain disrupted cable organization even during SDF1 chemotaxis (Fig. 4 D). Furthermore, they accumulate pMLC at E-cadherin junctions (Fig. 4), suggesting that contractility is stabilized at sites of cell–cell adhesion. This junctional remodeling is accompanied by a redistribution of FA components, such as p-PAX, from the periphery to the internal regions of the cluster (Fig. 7), indicating that adhesive signaling is redirected to support internally coordinated traction. These changes in cytoskeletal and adhesive organization result in a redistribution of force generation. TFM reveals that while mesenchymal clusters concentrate forces at the leading edge, epithelial-like clusters redirect traction internally, toward junctional regions (Fig. 8). This redistribution suggests that force transmission occurs through intercellular adhesions, consistent with previous work showing that epithelial monolayers migrate along axes of maximal tension via adherens junctions (Tambe et al., 2011). Interestingly, although protrusions are still present at the edge in epithelial-like clusters, their size increases during chemotaxis, becoming comparable with those in mesenchymal clusters (Fig. 5), and their contribution to traction force appears to cooperate with internal mechanics. These findings support the idea that mechanical coordination in epithelial collectives can emerge from the integration of junctional adhesion and spatially redistributed traction forces. Similar principles have been described in epithelial monolayers, where intercellular adhesions contribute to force propagation and coordination across the tissue (Bazellières et al., 2015; Serra-Picamal et al., 2012; Ladoux and Mège, 2017). Notably, cryptic protrusions within epithelial-like clusters become polarized during chemotaxis, further supporting a model of internal guidance that contributes to directional movement. This contrasts with the edge-dominated pulling observed in mesenchymal clusters and underscores an alternative mechanical mode of chemotaxis based on junctional coordination. The mechanical and adhesive properties of epithelial-like NC clusters resemble epithelial collectives observed in other biological systems. In mammary branching morphogenesis and carcinoma invasion, for instance, E-cadherin–mediated contacts are maintained during migration and contribute to polarity and coordination (Shamir and Ewald, 2015; Cheung et al., 2013). Similarly, in *Drosophila* border cell migration, E-cadherin plays a critical role in promoting directional movement through mechanical feedback across the cluster (Cai et al., 2014). In our system, sustained E-cadherin expression stabilizes both pMLC and p-PAX at junctions, providing an integrated platform where adhesion and contractility cooperate to support directional guidance. In contrast, mesenchymal NC clusters predominantly rely on N-cadherin for cell–cell interactions during migration, consistent with its established role in mesenchymal NC behavior (Theveneau and Mayor, 2012; Scarpa et al., 2015). Including N-cadherin in our model (Fig. 8 D) reflects this well-characterized adhesive context and highlights that distinct cadherin regimes support alternative collective migration strategies within the NC. This organization highlights a mode of migration that emerges from internal protrusive activity and intercellular coordination (Farooqui and Fenteany, 2005). Consistent with this, the redistribution of Rac1 activity observed in epithelial-like clusters further supports a shift in polarity mechanisms. In mesenchymal clusters, Rac1 localizes to the leading edge, consistent with leader-cell protrusions and classical front-rear polarity. In contrast, epithelial-like clusters show increased Rac1 activity at internal junctions aligned with the chemotactic gradient, although some peripheral activity remains. This suggests a partial internal polarity and supports a model in which collective guidance emerges not only from leader cells but also through coordinated junctional signaling across the cluster.

Importantly, the ability of NC cells to migrate collectively while retaining epithelial characteristics challenges the long-standing view that EMT and loss of E-cadherin are required for motility. Consistent with this, evidence from *Xenopus* demonstrates that E-cadherin function is required for proper NC migration (Huang et al., 2016). Supporting this idea, studies in chick embryos have shown that E-cadherin is robustly maintained during the early phases of cephalic NC migration and that its sustained expression limits mesenchymal dispersal (Dady et al., 2012; Rogers et al., 2013; Rogers et al., 2018). Our findings extend this by showing that, despite retaining high E-cadherin, epithelial-like NC clusters remain fully competent to undergo directed chemotaxis toward SDF1. While downregulation of E-cadherin has been associated with enhanced dispersion and invasiveness (Nieto, 2013; Lamouille et al., 2014), several studies have shown that E-cadherin–positive cells can also migrate collectively in a coordinated and efficient manner (Revenu and Gilmour, 2009; Campbell and Casanova, 2016). Our model provides direct experimental access to this phenomenon in the same organism, highlighting the plasticity of NC cells to adopt both epithelial and mesenchymal modes of migration depending on their adhesive context. This plasticity also reflects the heterogeneous organization of NC migratory streams, where epithelial and mesenchymal cells may coexist and cooperate during migration. Our results suggest that within the same migratory population, distinct mechanical strategies can operate in parallel or sequentially, depending on local interactions and guidance cues. This supports the growing recognition that epithelial and mesenchymal states exist on a continuum and that transitions between them are dynamic and reversible (Dongre and Weinberg, 2019). This view aligns with previous work showing that epithelial and mesenchymal migration modes are not strictly separated but represent dynamic and reversible states along a continuum. In both developmental and cancer contexts, hybrid or partial EMT states have been shown to support collective migration while preserving intercellular adhesion (Revenu and Gilmour, 2009; Campbell and Casanova, 2016; Peglion and Etienne-Manneville, 2024). Together, these observations highlight a broader biological plasticity that may benefit embryonic morphogenesis. The ability of NC cells to interpret chemotactic cues while retaining epithelial cohesion could help ensure coordinated tissue exit and preserve mechanical integrity at early stages of migration. This developmental flexibility raises several open questions for NC biology, including how junction-associated Rac1 activation is established before classical front-rear polarity, whether internal signaling contributes to maintaining stream stability, and how these mechanisms interact with EMT progression *in vivo*. More generally, our findings raise questions about whether other developmental collectives, such as placodes or mesodermal groups, use similar junctional signaling to integrate guidance cues before full EMT. Beyond development, this work also resonates with cancer systems, where hybrid EMT states drive collective invasion while maintaining E-cadherin–mediated junctions. The parallel suggests that internal guidance mechanisms, including junction-associated Rac1 activity, may contribute to clustered dissemination in metastasis and could represent potential targets for disrupting collective invasion.

Finally, by uncovering an alternative mode of collective chemotaxis in epithelial NC clusters, our work contributes to a broader understanding of how mechanical systems are reconfigured during guided migration. The NC model thus provides a powerful platform to dissect how adhesion, polarity, and contractility are integrated to enable robust and complex collective migration.

## Materials and methods

### *X. laevis* husbandry, fertilization, and embryo manipulation

Adult *X. laevis* were maintained and used under the regulations and guidelines of animal licenses assigned to this project by the UK Home Office and University College London. Ovulation of mature females was induced by intraperitoneal injection of 100 IU pregnant mare serum gonadotrophin (Intervet), followed 16 h later by 200–300 IU of human chorionic gonadotrophin (Intervet). Eggs were collected and fertilized *in vitro* by mixing them with a sperm solution diluted in 0.1× Marc’s Modified Ringer’s (MMR) solution. Embryos were maintained in 0.1× MMR at 14°C and staged according to Nieuwkoop and Faber (Nieuwkoop and Faber, 1958). In our case room temperature is 19°C.

### mRNA synthesis and microinjection and reagents

Embryos were de-jellied in 2% L-cysteine solution (pH adjusted with NaOH) and maintained in 0.1× MMR at 14°C until the appropriate developmental stage. To specifically label and manipulate cephalic NC cells, embryos at the eight-cell stage were injected using calibrated pulled glass needles into the two right animal ventral blastomeres in 4% Ficoll in 0.1× MMR. The left ventral blastomeres were left non-injected to serve as internal controls. Each injection volume was ∼5 nl. Embryos were incubated at 14°C in 0.1× MMR. For cell labeling and live tracking, embryos were injected with mRNAs encoding nuclear RFP (300 pg), membrane GFP (300 pg), and/or membrane mCherry (300 pg). To induce an epithelial-like phenotype, 800 pg of *Xenopus* E-cadherin mRNA were injected into each targeted blastomere. All mRNAs were synthesized *in vitro* using the mMESSAGE mMACHINE SP6 Transcription Kit (AM1340; Thermo Fisher Scientific) following the manufacturer’s instructions. mRNA quality and concentration were verified by agarose gel electrophoresis and spectrophotometry. Injected embryos were maintained in 0.1× MMR at 14°C until the required stage for dissection or imaging.

### NC grafting

For *in vivo* analysis of migratory behavior during early and late stages of NC migration, cephalic NC cells were grafted from fluorescently labeled donor embryos onto unlabeled host embryos. Donor embryos were injected at the eight-cell stage with H2B-RFP mRNA to label nuclei and cultured until stage 15–16. The cephalic NC territory was dissected under a stereomicroscope using eyebrow knives. Host embryos at the equivalent developmental stage were prepared by removing the corresponding NC region, and the labeled donor tissue was grafted into the same location, using anatomical landmarks for reproducibility. After transplantation, embryos were allowed to heal in 0.1× MMR at 14–18°C for at least 1 h before mounting for imaging. NC migration was monitored by time-lapse microscopy between St17 (early) and stage 25 (late), and nuclear trajectories were quantified using semiautomated tracking tools in Fiji. Graft placement and integration were confirmed by live fluorescence imaging and post hoc assessment of NC markers.

### Tissue fixation and cryosectioning

Embryos were fixed in MEMFA (0.1 M MOPS, pH 7.4, 2 mM EGTA, 1 mM MgSO_4_, and 3.7% formaldehyde) for 2 h at room temperature and washed extensively in PBS. Fixed embryos were bleached in 3% H_2_O_2_ in 1× SSC under bright light until pigmentation was reduced, rinsed in PBS, and cryoprotected overnight in 15% sucrose at 4°C. Embryos were then embedded in 15% sucrose and 30% fish gelatin, oriented to obtain transverse sections of the cephalic NC stream, frozen on dry ice, and cryosectioned at 30 µm thickness using a Leica CM3050 cryostat. Sections were mounted onto Superfrost Plus slides, dried briefly, and stored at −20°C until further processing for HCR or immunostaining.

### HCR and immunostaining on cryosections

Cryosections were brought to room temperature, rehydrated in PBS, and post-fixed for 10 min in 4% formaldehyde. Sections were equilibrated in HCR hybridization buffer (Molecular Instruments) for 5 min at 37°C and incubated overnight at 37°C with the twist probe set diluted in pre-warmed hybridization buffer. After hybridization, slides were washed in HCR wash buffer, equilibrated in amplification buffer, and incubated overnight at room temperature with snap-cooled fluorophore-conjugated hairpins (h1 + h2). Excess hairpins were removed by washing in 5× SSCT. Following completion of the HCR protocol, sections were permeabilized in 0.1% Triton X-100 for 10 min and blocked for 1 h in 10% normal goat serum (NGS) in PBS. Slides were incubated overnight at 4°C with mouse anti-E-Cadherin (5D3; DSHB, 1:50) diluted in blocking buffer. After washing in PBS, sections were incubated for 1 h at room temperature with Alexa Fluor–conjugated secondary antibodies (1:1,000), washed, and mounted. Images were acquired on a Leica SP8 confocal microscope using identical settings across samples, and Z-stacks were processed with Fiji.

### NC culture, chemotaxis, and dispersion assays

NC explants were dissected from embryos at St13 for epithelial-like clusters or St17 for mesenchymal clusters. Embryos were manually devitellinized in 1× DFA medium using fine tungsten needles. The chorion was removed, and the overlying epidermis was carefully dissected with a hair knife to expose the cephalic neural folds. Explants were excised using fine forceps under a stereomicroscope, then cut into small clusters using a hair knife, and immediately transferred to fibronectin-coated imaging dishes. NC identity was confirmed based on characteristic morphology and behavior *in vitro*, following established dissection criteria (Gouignard et al., 2020). For plating, μ-Dish 35-mm plates (high wall, Ibidi) were coated with 10 μg/ml fibronectin (Sigma-Aldrich) diluted in PBS and incubated for 1 h at 37°C. Explants were cultured in 1× DFA for the duration of the experiment.

For chemotaxis assays, acrylic heparin beads (Sigma-Aldrich) were incubated overnight at 4°C in 1 μg/ml SDF1 (Sigma-Aldrich) diluted in PBS. Glass-bottom dishes were coated with fibronectin (10 µg/m). A thin line of silicone grease was applied using a 20 μl syringe to delimit the culture area. SDF1-coated beads of similar diameter (150–200 µm) were selected with an eyebrow knife and aligned against the grease border in the fibronectin-coated area, spaced ∼1 mm apart. Excess beads were removed. NC explants were placed 250–500 µm away from the bead line and allowed to adhere for 30 min in DFA1X medium. Culture medium was added to fill the dish, and samples were imaged using water-immersion or inverted objectives.

For dispersion assays*,* NC explants were placed individually on fibronectin-coated dishes and allowed to migrate freely in DFA medium for 10 h. Time-lapse imaging was performed under the same optical conditions. Nuclear RFP signal was used to track individual cells and quantify the dispersion dynamics. To quantify dispersion, we applied the Delaunay triangulation algorithm (Carmona-Fontaine et al., 2011) using the corresponding ImageJ plugin. This method calculates the area of triangles formed between nearest-neighbor nuclei, and dispersion was inferred from the temporal increase in mean triangle area. Cell dispersion and cell migration toward the bead were recorded over 6 h by time-lapse microscopy using a Leica TCS SP8 confocal microscope with a 10× and 20× objective (Plan Fluotar 10×/0.30 NA) and DFC 300FX camera, controlled via LAS X software.

### Immunofluorescence

NC explants were fixed in 4% formaldehyde in PBS for 30 min at room temperature. Samples were permeabilized with 0.1% Triton X-100 in PBS for 10 min and blocked in 10% NGS. The following primary antibodies were used: anti-pMLC (ab2470; Abcam, 1:100), anti-Rac1-GTP (sc-514583; Santa Cruz Biotechnology, 1:500), anti-phospho-paxillin (pY118; Invitrogen, 1:500), and anti-E-cadherin (5D3; DSHB, 1:40). Alexa Fluor–conjugated secondary antibodies (A11008, A11001; Invitrogen, 1:500) and DAPI (20 µg/ml; D9542; Sigma-Aldrich) were used as counterstains. Samples were mounted in Mowiol and imaged using a Leica TCS SP8 confocal microscope with a 40× oil immersion objective under constant acquisition settings.

### Immunofluorescence during chemotaxis

NC explants were exposed to SDF-loaded beads as described above and allowed to migrate for 3 h. Following this, explants were fixed and processed for immunostaining as described in the previous section. Samples were stained for pMLC, E-cadherin, pPaxillin, Rac1-GTP, and DAPI. Imaging was performed using a Leica TCS SP8 confocal microscope with identical settings for all conditions.

### TFM

To measure traction forces generated by migrating NC clusters, polyacrylamide hydrogels of 600 Pa stiffness were prepared by adjusting the acrylamide and bis-acrylamide concentrations. Red fluorescent beads (0.5 μm diameter, Invitrogen) were embedded at the gel surface during the polymerization process. Gels were functionalized with 4 μM Sulfo-SANPAH (Thermo Fisher Scientific) under UV illumination and coated with 10 μg/ml fibronectin (Sigma-Aldrich) for 1 h at 37°C.

NC explants were plated on the gels in DFA medium and allowed to adhere. Imaging was carried out every 5 min using a Nikon AXR confocal microscope equipped with a 40× silicone immersion objective.

For traction force measurements during chemotaxis, acrylic heparin beads (Sigma-Aldrich) preincubated overnight at 4°C with 1 μg/ml SDF1 (Sigma-Aldrich) were embedded into the gels prior to cell seeding. NC explants were placed approximately two bead diameters away from the SDF1 source, as in standard chemotaxis assays. After live imaging, cells were removed by trypsinization, and relaxed bead positions were recorded. Traction forces were calculated by comparing bead displacements before and after cell removal using the Particle Image Velocimetry and Fourier Transform Traction Cytometry plugins in Fiji.

### Quantification of protrusive activity

To analyze protrusive activity in migrating NC clusters, cells were labeled with mbmCherry. Confocal z-sections were acquired at two distinct planes: one at the substrate level, highlighting basal protrusions, and a second plane above the substrate, highlighting cell bodies. The two planes were overlaid and pseudocolored (protrusions in cyan, cell bodies in magenta) to distinguish protrusive structures from the main cell body. Protrusions were defined as mbmCherry-positive extensions emerging from the substrate-facing membrane. Quantification was performed in Fiji by manually outlining individual protrusions using the freehand selection tool. For each cluster, protrusions were analyzed separately at the periphery and in internal regions.

### Quantification of E-cadherin fluorescence at cell–cell junctions

To quantify E-cadherin fluorescence at cell–cell contacts, a standardized line-scan method was applied using Fiji. In the E-cadherin channel, a straight line of ∼7 µm in length was drawn perpendicularly across the junction between two adjacent cells. This line was saved as a region of interest (ROI) and reused across all images to maintain consistent length and orientation between samples. Fluorescence intensity along the line was extracted using the “Plot Profile” function, generating intensity values at ∼0.3-µm intervals. Values were exported and analyzed in Excel. For each cluster, the average intensity profile was calculated from multiple junctions. The point of maximum fluorescence intensity along the line was defined as the center of the junction (position 0). For normalization, the peak junctional intensity was divided by the average fluorescence in adjacent cytoplasmic regions.

### Protrusion orientation analysis

The orientation of each protrusion was determined by measuring the angle of the vector between the centroid of the cell and the centroid of the protrusion, using ImageJ. Measurements were used to construct polar plots of directional protrusive activity.

### Fluorescence intensity quantification

For each experimental condition (mesenchymal and epithelial-like), fluorescence intensity was quantified in two defined regions within each cluster: the periphery and the internal area. ROIs were manually selected using Fiji based on the cluster geometry and signal localization. The mean fluorescence intensity (arbitrary units) was calculated separately for each region in individual z-projected images. For each cluster, the total fluorescence (peripheral + internal) was used to normalize each value and express fluorescence distribution as a percentage of the total.

### Statistical analysis

Statistical analysis was carried out using Prism9 (GraphPad). One-way ANOVA followed by Tukey’s multiple comparisons posttest was used for multiple group comparisons. Unpaired two-tailed Student’s *t* tests were applied for two-group comparisons. The type of statistical test and exact sample size (*n*) are specified in the figure legends. Significance was defined as follows: ****P ≤ 0.0001; ***P ≤ 0.001; **P ≤ 0.01; *P ≤ 0.05; n.s., not significant.

### Online supplemental material

Fig. S1 Shows the progressive increase in cell dispersion and the gradual decline in junctional E-cadherin across St13, Stage 15, and St17 NC explants plated on fibronectin. Fig. S2 presents E-cadherin levels in St13, St17, and St17 + E-cadherin NC clusters and further shows that E-cadherin remains stable during the chemotactic window in St13 explants. Fig. S3 shows how E-cadherin disrupts the peripheral actomyosin cable in mesenchymal clusters and redirects pMLC toward junctional regions in epithelial-like clusters during SDF1 chemotaxis. Fig. S4 shows the spatial distribution of traction forces in mesenchymal and epithelial-like clusters in the absence of chemotactic cues. Video 1 shows the gradual transition in cell dispersion across St13, Stage 15, and St17 NC explants. Video 2 compares the cell dispersion of St13, St17, and St17 + E-cadherin clusters plated on fibronectin. Video 3 shows chemotactic responses of NC clusters exposed to SDF1. Video 4 shows internal cryptic protrusions in the absence of external cues. Video 5 shows polarization of internal protrusions in epithelial-like clusters during chemotaxis.

## Data Availability

All data are available in the main text or the supplementary materials.
